# Three‐dimensional differences in plantar surface shape captured by methods used for custom accommodative insole design

**DOI:** 10.1002/jfa2.70034

**Published:** 2025-01-30

**Authors:** Kimberly A. Nickerson, Christina Carranza, Scott Telfer, William R. Ledoux, Brittney C. Muir

**Affiliations:** ^1^ VA RR&D Center for Limb Loss and MoBility (CLiMB) VA Puget Sound Health Care System Seattle WA USA; ^2^ Department of Mechanical Engineering University of Washington Seattle WA USA; ^3^ Department of Orthopaedics and Sports Medicine University of Washington Seattle WA USA

**Keywords:** 3D scanning, custom accommodative insoles, diabetes, plantar surface shape

## Abstract

**Background:**

The patient‐specific shape of custom accommodative insoles for individuals with diabetes provides full foot‐to‐insole contact, offloading areas with high plantar pressures and reducing ulceration risk. To design the insole surface, plantar surface shape is captured, traditionally with a foam crush box impression or more recently with 3D scans of the foot. Beyond discrete measurements of the foot, the overall plantar surface shapes obtained from these different methods have yet to be compared, however, differences in the shapes captured by these methods may affect the insole's surface geometry design and subsequent performance.

**Methods:**

Plantar surface shapes of 12 individuals with diabetes were captured using a foam crush box, flatbed 3D foot scanner, and handheld 3D scanner. Foot length, width, arch height, and arch volume were measured from each shape‐capture method and compared. Mesh‐to‐mesh distances between the foam crush box mesh and the direct scanning method meshes for each subject were calculated.

**Results:**

Foot length and width measured from the foam crush box scan were greater than the foot length measured from the flatbed scan and handheld scan. The flatbed scan also measured a length and width greater than the handheld scan. Arch heights and volumes from the flatbed scan were less than the heights calculated from the foam crush box and handheld scan. Mesh‐to‐mesh distances for the flatbed scan and areas of the foot not in contact with the scanner were inferior to the corresponding areas in the foam crush box impression. For the handheld scan, the lateral hindfoot and midfoot were superior, and the medial forefoot was inferior to the foam crush box impression.

**Conclusions:**

Different clinical methods used to capture foot shapes for the design of accommodative insoles may result in different plantar surface shape outputs and therefore impact custom accommodative insole design.

## 1 INTRODUCTION

Diabetes is a leading cause of lower limb amputation in the United States [1]. Eighty‐four percent of lower limb amputations in individuals with diabetes are preceded by ulceration as peripheral neuropathy, tissue changes, and high plantar pressures associated with a diagnosis of diabetes contribute to high ulceration risk in this population [2, 3]. A common nonsurgical intervention to reduce risk of ulceration and subsequent amputation in individuals with diabetes is the prescription of custom accommodative insoles designed to reduce areas of elevated plantar pressures [4, 5, 6]. Standard‐of‐care custom accommodative insoles are manufactured from multi‐layer foam and are shaped to conform to an individual's foot [7, 8]. The patient‐specific shape of custom accommodative insoles provides full foot‐to‐insole contact, which redistributes loads from areas of high plantar pressures to other areas of the foot [9, 10].

Patient‐specific conformity between a custom accommodative insole and the plantar surface of the patient's foot is traditionally achieved by creating a positive mold of the patient's plantar surface from which the multi‐layer foams are formed. Several methodologies can be used to collect patient plantar surface shapes for the design of accommodative insoles [11]. Currently, the standard shape‐capture method is a seated partial weight‐bearing foam crush box impression (a single‐use foam tray used to obtain a negative partial weight‐bearing impression of the plantar surface of the foot) [12]. However, recent advances in 3D scanning have led to this approach being used to capture the foot shape, either via a flatbed or handheld scanners [13, 14, 15]. For both foam crush boxes and flatbed and handheld scanning devices, evaluations of intra‐rater reliability of basic measures of foot shape (i.e., foot length and width) have been conducted and demonstrated moderate to excellent ratings [16, 17, 18]. Differences in foot length and width measured from foam crush box impressions versus 3D scanning have been found and may be used to explain differences in the overall scaling of insoles produced from various methods. However, these metrics (foot length and width) provide limited information on how the overall surface shape and contour of the insole may differ [12, 19]. Despite 3D scanning implementation in clinical settings for the production of insoles, a more comprehensive comparison of overall plantar surface shapes captured by each method has yet to be conducted.

Arch volume and mesh‐to‐mesh distance (distances between the virtual rendition of two surfaces) calculations have been developed and used to assess plantar surface shape in various applications but have not yet been used to evaluate the differences in plantar surface shape captured by 3D scanning and foam crush box impressions [20, 21]. Because of the importance of arch support and plantar surface conformity to the pressure‐reducing function of accommodative insoles, evaluating these metrics for foam crush box impressions and scanning may be important to understanding the respective insole function. The purpose of this study is to evaluate 3D plantar surface shape, in addition to basic measures of foot shape such as foot length and width, captured by 3D scanning and foam crush box impressions. The results of this study may be used to inform clinical procedures for plantar shape capture used during accommodative insole design and fabrication.

## 2 MATERIALS AND METHODS

**FIGURE 1:**
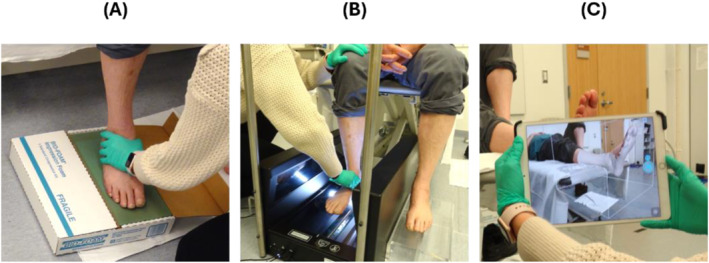
Capture of foot shape by (A) foam crush box, (B) flatbed scanner, and (C) handheld scanner.

Twelve individuals (71 ± 7 years, 10 Males) with diabetes and a current custom accommodative insole prescription were recruited to participate in this study. All participants provided written informed consent and all study protocols were approved by the VA Puget Sound institutional review board prior to data collection. The shape of each participant's right and left foot was captured by a certified orthotist/prosthetist using a single scan from each of the three following foot shape‐capture devices: (1) a foam crush box (Bio‐Foam Foot Impression Box (Kent, OH, USA)), (2) a flatbed scanner (Tiger ® 3D Foot Scanner (Go 4‐D Inc., ON, Canada)), and (3) a handheld scanner (Structure Sensor for iPad (XRPro LLC (Structure), MI, USA)). During the foam crush box impression and scan with the flatbed scanner, participants were seated, whereas the orthotist held their foot in a subtalar neutral position. For the scan taken with the handheld scanner, the participant lay in a supine position with their foot relaxed at the end of the examination table (Figure 1).

**FIGURE 2:**
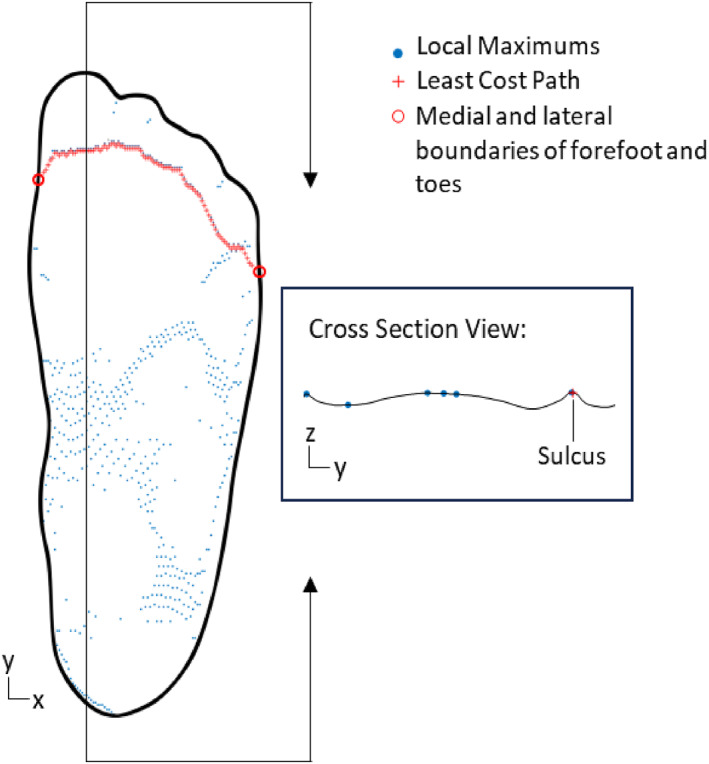
The least cost path along the sulcus beneath the toes identified from local maximums in y–z cross‐sections.

For each participant, the plantar surface region of the scans obtained from the flatbed and handheld scanners were compared to the plantar surface shape obtained from the foam crush box impression. For digital comparison of the foot shape resulting from all three capture methods, the foam crush box impression was also scanned (Creaform, Quebec, Canada). Details for each scanner are provided in Table 1 following the consistent reporting three‐dimensional scanning (CRITIC) checklist to support transparency and reporting consistency between studies [15].The scans from all three methods were imported into Rhino ® (V7; Robert McNeel & Associates, WA, USA) to be aligned and cropped. First, scans were aligned to simulate the position of a foot in a Brannock device. A series of axis–angle rotations were performed so that manually selected points on the heel and first and fifth metatarsal heads were aligned with the ground plane. As each method captured differing amounts of the foot, each mesh was cropped to isolate the plantar surface excluding the toes. To define the plantar surface region, mesh triangles with a surface normal vector angled 30° or greater below the ground plane were identified [22]. The curve defining the boundary between plantar surface mesh triangles and nonplantar surface mesh triangles was smoothed and used to split the mesh. At the boundary of the forefoot and toe region, the mesh of the plantar surface region was trimmed further. First, to identify the boundary between the forefoot and toe region, the points along the sulcus beneath the toes were identified as local maximums in sagittal plane cross‐sections of the foot. To create sagittal plane cross‐sections of the foot, a uniform grid with 1‐mm spacing spanning the width (*x*‐axis) and length (*y*‐axis) of the mesh was created in MATLAB (The MathWorks Inc., MA, USA). Z‐coordinates (height) of the mesh were interpolated at each *x*–*y* coordinate of the grid. From the interpolated z‐values uniformly spaced in *x* and *y*, for each sagittal plane cross‐section of the grid local maximums were identified as possible coordinates along the sulcus. A cost function where the least cost path algorithm is minimized at these possible sulcus coordinates was used to identify the sulcus curve between two manually identified points (the most medial and lateral point of the sulcus) [23]. This curve was smoothed and used to trim the mesh (Figure 2).

**TABLE 1:** Consistent reporting three‐dimensional scanning (CRITIC) checklist for each shape‐capture method.

		Foam crush box/Scanner	Flat‐bed scanner	Handheld scanner
Participants	Sample size	12	12	12
	Men/Women	10,2	10,2	10,2
Equipment	Scanner model	Bio‐foam foot impression box (Kent, OH, USA), Go!SCAN 50 scanner (Creaform, Quebec, Canada)	Tiger ® 3D foot scanner (Go 4‐D Inc., ON, Canada)	Structure Sensor for iPad (XRPro LLC (Structure), MI, USA)
	Scanner type	Structured white light (scanner)	Optical laser scanner	Class 1 laser Projector
	Scanner Resolution	0.5 mm (scanner)	0.5 mm (standard deviation)	1040 × 1200 (max) depth 1080 × 1200 (max) IR
	Scanner Accuracy	0.1 mm (scanner)	Not found	1 mm
	Scanner capture duration (s)	≥ 120 s (box), 120 s (scanner)	5–15 s	0–120 s
	Scanner Calibration methods	Calibration plate w/white background and specifically placed targets	Not found	(1) Pollock Calibration—uses indoor calibration target (paper with pattern), see target in both IR and visible frames, tap “Got it!”, and proceed with refinement stage (2) find scene with clearly defined edged and manually adjust colored image to match edges, tap “save Calibration”
	Scanner reliability	NA	0.9–1.2 mm	NA
	Markers used	NA	NA	NA
	Number of scans	1	1	1
Participant Condition	Weight‐bearing	No (Sitting)	No (Sitting)	No
	Barefoot/Socks	Barefoot	Barefoot	Barefoot
	Unilateral/Bilateral scanning	Bilateral	Bilateral	Bilateral
Measurements	Foot measures used	Plantar surface (foot length, width, arch height, arch volume, and mesh‐to‐mesh distance)	Plantar surface (foot length, width, arch height, arch volume, and mesh‐to‐mesh distance)	Plantar surface (foot length, width, arch height, arch volume, and mesh‐to‐mesh distance)
Post Processing	Software used	Rhino ® (V7; Robert McNeel & Associates, WA, USA), Creaform VXElements (Creaform, Quebec, Canada), MATLAB (The MathWorks Inc., MA, USA), and 3D Slicer's SlicerMorph module	Rhino ® (V7; Robert McNeel & Associates, WA, USA), MATLAB (The MathWorks Inc., MA, USA), and 3D Slicer's SlicerMorph module	Rhino ® (V7; Robert McNeel & Associates, WA, USA), MATLAB (The MathWorks Inc., MA, USA), and 3D Slicer's SlicerMorph module

**FIGURE 3:**
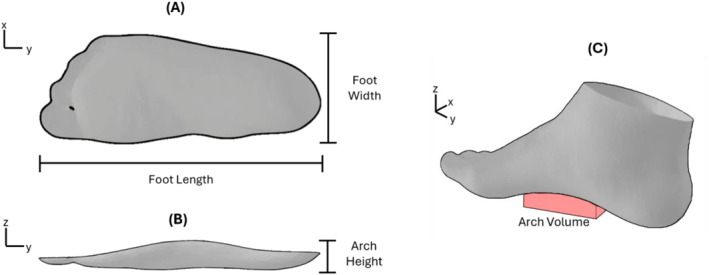
Schematic of foot measurements. (A) Foot length and foot width, (B) arch height, and (C) arch volume.

**FIGURE 4:**
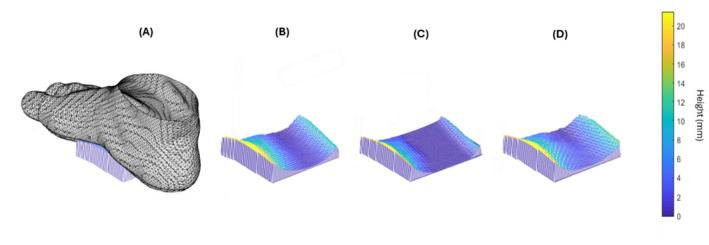
Example arch volumes. (A) Arch volumes from a single representative subject's, (B) foam crush box impression, (C) flatbed scan, and (D) handheld scan.

Shape parameters for each aligned and cropped mesh were computed to compare the plantar surface shapes obtained from the foam crush box method to the shapes from the scanning methods for each participant's right and left foot. Traditional one‐dimensional measurements of foot length, width, and arch height were measured from the meshes (Figures 3A and 3B) [22]. Foot length and width were measured as the largest distance between two mesh vertices along the *y*‐axis and *x*‐axis, respectively. The largest distance along the *z*‐axis between a mesh vertex and the ground plane in the midfoot region (middle third of the mesh) was taken as the arch height. Additionally, more comprehensive measures, arch volume and mesh‐to‐mesh distances, were calculated for each mesh. Arch volume was calculated as the volume between the plantar surface of the foot and the ground plane in the middle third of the foot (Figure 3, 4) [24]. Mesh‐to‐mesh distances for each participant's right and left foot were calculated as the vertical distances between corresponding landmarks on the foam crush box impression and direct scanning meshes. A multiple template 3D elastic matching algorithm implemented in 3D Slicer's SlicerMorph module [25, 26] was used to place landmarks on each mesh creating landmark correspondence with the template meshes [27, 28]. SlicerMorph was also used to choose template meshes and perform the initial landmarking, with 1480 landmarks, on the template meshes [29].

Descriptive statistics (mean and standard deviation) were calculated for foot shape parameters of foot length, foot width, arch height, and arch volume. Differences in these metrics between the foam crush box impressions and the two direct scans were assessed with a repeated measures ANOVA with a within‐subjects factor of foot shape‐capture method. Bonferroni corrections were applied to an initial *α* of 0.05 to correct for multiple comparisons. Effect sizes were quantified with eta squared (*η* ^2^) for repeated measures ANOVA. Mean mesh‐to‐mesh distances between the foam crush box scans and each of the direct scanning methods were calculated for each unique landmark. A one‐sided *t*‐test on the distances between the foam crush box scans and each scanning method was used for each landmark. A false discovery rate correction of *q* = 0.05 was used to account for the large number of comparisons.

## 3 RESULTS

### 3.1 Foot length, foot width, and arch height

Foot lengths measured from the foam crush box scan, flatbed scan, and handheld scan were not significantly different from each other (*p* = 0.054; Table 2). The effect size, as measured by eta squared (*η* ^2^), was 0.07, which is considered a small effect size, indicating that a small amount of the total variability is explained by the shape‐capture methods. The trends indicate that foot length measured from the foam crush box scan was greater than the foot length measured from the flatbed scan and handheld scan. The flatbed scan also measured a length greater than the handheld scan. Foot width measured from the foam crush box scan, flatbed scan, and handheld scan were significantly different from each other (*p* = 0.007). The effect size, as measured by eta squared (*η* ^2^), was 0.13, which is considered a medium effect size, indicating that a considerable amount of the total variability is explained by the shape‐capture methods. Foot width measured from the foam crush box scan was significantly greater than the flatbed scan (*p* = 0.03) and handheld scan (*p* < 0.001) and the width measured from the flatbed scan was also greater than the handheld scan (*p* < 0.001). Arch height measured from the foam crush box scan, flatbed scan, and handheld scan were significantly different from each other (*p* < 0.001). The effect size, as measured by eta squared (*η* ^2^), was 0.18, which is considered a large effect size, indicating that a large amount of the total variability is explained by the shape‐capture methods. Arch heights calculated from the flatbed scan were significantly less than the heights calculated from the foam crush box (*p* = 0.002) and handheld scan (*p* < 0.001). There were no significant differences in arch height between the foam crush box and the handheld scan.

**TABLE 2:** Mean (SD) foot shape parameters for each capture method.

Dimension	Foam crush box	Flatbed scanner	Handheld scanner	*p*‐value	(*η* ^2^)
Foot length (cm)	26.9 (1.6)	26.2 (1.7)	25.8 (1.9)	=0.054	0.07
Foot width (cm)	9.95 (.66)	9.81 (.60)^a^	9.39 (.62)^a^ ^+^	=0.007	0.13
Arch height (cm)	2.80 (.59)	2.80 (.59)	2.98 (.44)^+^	<0.001	0.18
Arch volume (cm^3^)	61.8 (20.1)	32.2 (11.4)^a^	32.2 (11.4)^a^	<0.001	0.45

^a^ Significantly different from the foam crush box impression.

^+^ Handheld scanner is significantly different from flatbed scanner.

### 3.2 Arch volume and mesh‐to‐mesh distance

Arch volume measured from the foam crush box scan, flatbed scan, and handheld scan were significantly different from each other (*p* < 0.001). The effect size, as measured by eta squared (*η* ^2^), was 0.45, which is considered a large effect size, indicating that a large amount of the total variability is explained by the shape‐capture methods. Arch volumes calculated from the flatbed scan were significantly less than the volumes calculated from the foam crush box (*p* < 0.001) and handheld scan (*p* < 0.001). There were no significant differences in the arch volume between the foam crush box and handheld scan (Table 2).

**FIGURE 5:**
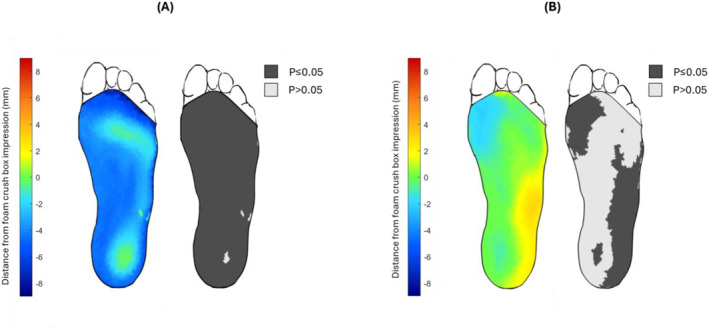
(A) Color map of the average vertical distances and significant differences in vertical mesh positioning between (A) the foam crush box impression and the flatbed scanner, and (B) the foam crush box impression and the handheld scanner. A negative distance indicates that the scan position is inferior to foam crush box. Dark gray indicates areas of significant differences (*p* ≤ 0.05, *q* ≤ 0.05).

The average vertical mesh‐to‐mesh distances between the foam crush box impressions and the scanning methods for all subjects are represented as color maps (Figure 5). For the flatbed scan, areas of the foot not in contact with the scanner were inferior to the corresponding areas in the foam crush box impression. For the handheld scan, the lateral hindfoot and midfoot were superior, and the medial forefoot was inferior to the foam crush box impression. Areas with significant differences (*p* < 0.05 and *q* < 0.05) in average landmark position between the foam crush box impressions and scans include the plantar surface, except the bottommost part of the heel for the flatbed scan and the lateral hindfoot and midfoot and medial forefoot for the handheld scan.

## 4 DISCUSSION

The foot shape captured during the fabrication process of custom accommodative insoles is used to design the surface of an insole to match the contours of the plantar surface, creating conformity to redistribute and reduce high plantar pressures [4, 5, 6]. This study is a preliminary investigation comparing plantar surface shape outputs from different clinical methods of obtaining foot shape. The results of this study indicate that shape‐capture methodology (including device, foot posture, and weight‐bearing) impacts the captured plantar surface morphology which could subsequently affect the design and function of custom accommodative insoles.

### 4.1 Foam crush box impression

Foam crush box impressions exhibited the largest foot length and width measurements of the three plantar surface shape‐capture methods. Foot movement during the impression process likely contributed to the greater length and width measurements seen with the foam crush box impressions. For example, slight movement of the foot in the anterior/posterior and mediolateral directions while removing the foot vertically from the foam could result in extra displacement of the foam and an impression longer/wider than the actual foot. Laughton et al. similarly reports forefoot width measured from a foam crush box to be larger than widths measured from a seated flatbed scan [30].

### 4.2 Flatbed scan

The flatbed scans produced arch measurements that were significantly less than the measurements taken from the foam crush box impressions or handheld scans. The partial weight‐bearing position of the foot against the rigid glass surface of the flatbed scanner surface likely contributes to these differences in arch height and arch volume as the soft tissues on the bottom of the foot are compressed [31, 32]. The greater compression of the soft tissues during the flatbed scans is demonstrated by the vertical mesh‐to‐mesh distances between the flatbed scans and foam crush box impressions. For almost all locations on the plantar surface (besides the lowest points on the heel and forefoot that have contact with the ground plane), the flatbed scans are on average lower than the foam crush box impressions revealing a different surface shape, and specifically a flatter, lower, less pronounced arch.

### 4.3 Handheld scan

The handheld scans produced significantly smaller foot length and width measurements than the foam crush box impression and flatbed scanner plantar surface shape‐capture methods. The difference in foot and body positioning during the handheld scan compared to the flatbed scan and foam crush box impression likely affected these foot measurements. The flatbed scans and foam crush box impressions are taken in partially weight‐bearing positions as the orthotist applies some downward force to hold the foot in a subtalar neutral position, which may result in some splaying of the plantar surface. The foot during the completely nonweight‐bearing handheld scan will not experience the same splaying effect and therefore may have a smaller measured length and width [31]. The medial–longitudinal arch shape captured by the handheld scans was similar to the foam crush box, however, significant differences were found in the vertical position of the lateral foot mid and hindfoot. This variation in lateral plantar surface height may also be attributed to the difference in foot posture between the supine and seated captures. The foot is not held in subtalar neutral during the handheld scan, allowing the foot to rest in an everted position.

### 4.4 Implications for insole design

The addition of arch volumes and mesh‐to‐mesh distance metrics presented in this paper provides more detailed information on how the insoles resulting from these shape‐capture methods may differ when compared to the existing body of work looking at shape‐capture methods with traditional measurements of foot length, width, and arch height [12, 33, 34]. Arch volume and mesh‐to‐mesh distances were used to visualize and quantify the differences in surface shape of insoles produced from these various methods that cannot be captured by discrete length, width, and height measurements. The lower less pronounced arch shape captured by the flatbed scanner in this study may indicate that insoles designed with a flatbed scan shape‐capture method may have reduced arch support and therefore decreased plantar pressure redistribution compared to insoles designed with a foam crush box impression [35]. Additionally, insoles designed from a handheld scan may not be the appropriate shape to hold the foot in a proper subtalar neutral alignment [19]. Including metrics comparing the plantar surface shapes used to generate insole surfaces from the different shape‐capture methods may be essential to understanding any differences in the performance of the resulting insoles.

### 4.5 Limitations

This study has some limitations. First, the shape‐capture methods investigated in this study do not represent the numerous variations in shape‐capture techniques used by clinicians globally. For example, different clinicians may choose to position the foot differently during a foam crush box impression or scan. We chose the shape‐capture methods used in this study with input from our clinical team to best represent the most standard practices in the orthotics field. Additionally, as this is a preliminary study into the three‐dimensional differences in shape‐capture techniques, the methods developed to crop, align, and compare the meshes obtained from the different shape‐capture techniques are novel and their full utility should be independently assessed. Future exploration may be necessary to establish a workflow to assess three‐dimensional differences in foot shape. Finally, in Table 2 we conducted a group mean comparison, as opposed to evaluating differences by individual, this could mask individual effects, as with our proposed future work, we could reevaluate this analysis.

## 5 CONCLUSIONS

We have demonstrated that different clinical methods used to capture foot shapes for the design of accommodative insoles may result in different plantar surface shape outputs. As plantar surface shape is used to design an insole's surface geometry, clinicians should be aware that foam crush box impressions and scanning devices may produce different outputs. While it is still uncertain how these differences in shape output may affect the design of insoles and their ability to reduce plantar pressures, the results of this study justify future work evaluating the performance of insoles developed from various shape‐capture methods.

## AUTHOR CONTRIBUTIONS

**Kimberley A. Nickerson:** Data curation; formal analysis; investigation; methodology; software; writing—original draft. **Christina Carranza:** Investigation; methodology; writing—reviewing and editing. **Scott Telfer:** Funding acquisition; supervision; reviewing; writing—reviewing and editing. **William R. Ledoux:** Funding acquisition; supervision; reviewing; writing—reviewing and editing. **Brittney C. Muir:** Conceptualization; funding acquisition; investigation; methodology; project administration; supervision; writing—reviewing and editing.

## CONFLICT OF INTEREST STATEMENT

The authors declare no conflicts of interest.

## ETHICS STATEMENT

The study was approved by the Department of Veterans Affairs Institutional Review Board (1588070).

## Data Availability

Research data are not shared.
